# Straining at Work and Its Relationship with Personality Profiles and Individual Consequences in Healthcare Workers (HCWs)

**DOI:** 10.3390/ijerph17020610

**Published:** 2020-01-17

**Authors:** Tiziana Ramaci, Massimiliano Barattucci, Francesca Vella, Paola Senia, Emanuele Cannizzaro, Alessandro Scorciapino, Caterina Ledda, Andrea De Giorgio, Venerando Rapisarda

**Affiliations:** 1 Faculty of Human and Social Sciences, University of Enna “Kore”, 94100 Enna, Italy; tiziana.ramaci@unikore.it; 2 Faculty of Psychology, e-Campus University, 22060 Novedrate (CO), Italy; massimiliano.barattucci@uniecampus.it (M.B.); andrea.degiorgio@uniecampus.it (A.D.G.); 3 Occupational Medicine, Department of Clinical and Experimental Medicine, University of Catania, 95121 Catania, Italy; francescav.89@libero.it (F.V.); paosenia@hotmail.it (P.S.); vrapisarda@unict.it (V.R.); 4 Department of Sciences for Health Promotion and Mother and Child Care “Giuseppe D’Alessandro”, University of Palermo, 90127 Palermo, Italy; emanuele.cannizzaro@unipa.it; 5 Department of Prevention, Area of Protection and Safety in the Workplace, Provincial Health Authority, 95124 Catania, Italy; alescorc@gmail.com

**Keywords:** straining, personality, health consequences, HCWs, stress at work

## Abstract

Straining is an attenuated form of mobbing, in which the continuity of vexatious actions is not driven by a discriminatory intent. With the objective of testing the possible moderating role of personality in the relationship between perceptions about straining at work and individual consequences, a correlational design research involved 374 healthcare workers (HCWs) from two Italian hospitals. The following questionnaires were administered: (1) Short Negative Acts Questionnaire (S-NAQ), to assess discriminative actions at work); (2) the Italian version of the Big Five Inventory (BFI-10 scale), to assess personality factors; (3) Occupational Stress Indicator (OSI), to measure psychological and physical health. Regression analysis and Structural Equation Models (SEM) were computed in order to test the relationships between variables. Perceived straining showed significant correlations with both psychological and physical health. Conscientiousness was inversely proportional to work-related bullying (WB), as agreeableness was to personal bullying (PB). Emotional stability was negatively correlated with all the three component scales of S-NAQ: WB, PB, and social bullying. The results seem to confirm that straining perceptions especially elicit, through emotional stability, psychological consequences. As regards the role of emotional stability in risk perceptions, it seems management has to pay thorough attention to personal factors in organizational perceptions and to straining actions.

## 1. Introduction

Changes in the labor market, evolution of production processes, new technologies, and competitiveness on a global scale have significantly transformed the work environment and demands on workers [1,2]. Some of these transformations may be associated with negative consequences on the psychophysical health of workers [3].

Literature on the dysfunctions of work organizations and their subsequent degenerative manifestations is huge, and it is therefore difficult to find a way amongst clearly vexatious phenomena such as mobbing or bullying and stress situations that are rather related to strategic, environmental, or relational conditions [4].

Judicial references [5] as well as literature references [6,7] seem to clearly indicate the need to pay attention to a series of work situations that are detrimental to the dignity of workers (demotion, de-skilling, isolation, degenerated conflicts, etc.) [8] and that are currently classified as straining and not attributable to other degenerative phenomena.

Workplace bullying is the systematic, repetitive, and intended undesirable behavior of one employee or group of employees targeting another employee or group of employees. Such behavior might consist of public humiliation and condemnation, social segregation, verbal exploitation, intimidation, inaccurate allegations, ignoring someone for a long period of time, and repeated reminders of someone’s errors. Workplace bullying is also referred to as abuse in the workplace, mobbing, harassment, and mistreatment. Often, bullying is a reflection of a systemic malfunction of a given institution rather than of direct personal animosities. Considering the spread of the phenomenon and the adverse effects it generates for both victim employees and employer, research on workplace bullying is thriving [9,10,11].

The focus of institutions and the scientific community on occupational health and safety is progressively increasing, leading to a continuous regulatory evolution and the development of good practices in safety and prevention, with the aim of reducing costs for businesses and the society.

Within this frame, this study aimed to explore the impact that perceptions of being a victim of straining actions at work have on individual consequences and the role of personality in the interpretation of negative actions and in its impact on a person.

### 1.1. Straining between Stress and Mobbing

The term straining derives from the verb “to strain” which means “to become stretched, to experience pressure, or to make something do or experience this” and refers to a phenomenon of both psychological and legal value, mainly resulting from an organizational conflict [12]. Straining is a psychological condition halfway between mobbing and simple occupational stress [10]: it is in fact a type of “forced” stress, i.e., higher than that related to the nature of work and targeting a victim (or a group of victims) in an intentional and discriminatory way, so as to cause a permanent worsening of the working condition of the worker, even before impacting his/her psychophysical health [13,14].

Straining is an attenuated form of mobbing, in which the continuity of vexatious actions, deliberately imposed by the hierarchical superior, does not show a discriminatory intent [12]. The interest in straining stems from the need to give a precise name and a specific compensation profile to situations of work discomfort, which nevertheless risk being unfairly bypassed as they do not fall within the definition of mobbing [12].

For a conduct to be defined as straining, even a single action is sufficient, provided its effects are long-lasting, as in the case of demotion or relocation [14,15,16,17].

The victim of straining is at a disadvantage compared to the strainer and must have suffered at least one negative action that has an effect at the occupational level in the long term and in a constant manner [18,19].

In addition to the affinities with mobbing, the term straining is also very close to the verb “to stress”: in a situation of straining, the aggressor will systematically tend to make his/her victim fall into a specific condition of stress; in this case, the type of stress could be defined as higher than the stress of work and of normal organizational interactions [20,21].

Therefore, and as initially said, straining, which according to some interpretations would be placed halfway between occupational stress and mobbing, in some situations becomes a prodromal phenomenon of mobbing [18,22]. When we talk about straining, we can distinguish different protagonists: as in mobbing, there are actors, victims, and spectators [13].

In the literature, overall, straining is associated both with forms of occupational stress due to poor work organization and with forms of forced stress resulting from discriminatory acts [23,24,25,26].

### 1.2. Negative Actions at Work and Personality

Numerous papers have sought to investigate organizational and role factors that are related to adverse health outcomes and other occupational outcomes [27,28,29,30]. Part of the literature has focused instead on individual variables and personality traits in order to understand if and how the worker’s personality could affect the perceived negative actions and the impact these have on well-being [31,32,33,34,35,36,37,38].

Some studies, on the other hand, have focused on the exploration of personality characteristics associated with the role of victim or mobbing agent [31], such as, for instance, personality disorders: paranoid, narcissistic, borderline traits would be more likely associated with the mobber profile; vice versa, profiles of whining, sad, rigid, prickly personality would be more frequently associated with people who perceive themselves as victims of mobbing or straining [20,39,40].

Overall, the contribution of mobbing researches does not seem to clarify the personality differences between victims and non-victims [41]. In fact, if on the one hand some personality traits (anxiety, depression, somatization, etc.) are more frequently found in victims of mobbing [36,38,42,43,44,45], other studies show a rather limited role of personality characteristics in situations of conflict at work [29,30,46,47,48].

As far as straining is concerned, some indications point out that it can have strong negative effects on the victim’s personality and, more specifically, on conscientiousness, friendliness, and open-mindedness [38]. The victim’s personality traits, therefore, could represent the outcomes of negative actions perceived at work rather than the causes of the harassment suffered [49,50,51].

Although some studies have focused on the contribution and role of personality factors in relation to mobbing actions’ effects on health consequences, very few researches have explored the effects of straining, mediated through personality, on HCWs.

### 1.3. Negative Actions and Healthcare Workers

Healthcare works (HCWs) are one of the categories of workers that are most at risk of work-related stress [52]. According to the survey conducted by the European General Practice Research Network (2019) on a group of 100,000 doctors operating in 12 countries, Italian doctors showed stress rates (43%) almost twice as high as the average stress rate of their European colleagues (22%).

The Medscape Lifestyle Report, produced by the Medscape National Physician Burnout, Depression & Suicide [53] scientific portal, focused primarily on burnout and depressive symptoms and on how they affect the activity of HCWs and their approach to the patient. In this report, 15,000 U.S. white coats from 29 different specializations were interviewed, and 50% stated that burnout significantly affects the quality of care offered to patients.

This is the case of “hostile or sporadic discriminatory actions, lacking the continuity requirement, and exerting effects that are continuous in time”. Among these: “groundless deprivation of work tools; assignment of tasks that are not compatible with the personal condition of the worker; unjustified displacement to a distressing site; underestimation of the work delivered by the worker”.

### 1.4. The Present Research

Reviewing the literature, on the one hand there is a definite need to more accurately investigate the effect of straining actions (in terms of duration and intensity) on the psychophysical health of workers; on the other, the need to test the role of personality factors in the interpretation of negative actions and in its impact on the individual [41].

Straining is an attenuated form of mobbing that does not show continuity of vexatious actions. However, actions not only are stressful but also lead to enduring and constant consequences that may indeed cause serious psychosomatic, psychophysical, or psychic disorders to the worker. Straining intentionally targets one or more people in a discriminatory way, and the victim suffers at least one action exerting a negative effect on his/her work environment. The worker, in a constantly inferior position in relation to the strainer, is subject to hostile actions, though limited in number and distant in time (i.e., not tout court compliant with mobbing parameters), that produce a negative, constant, and permanent change of the work situation and may likely affect the right to health, as stated by law. Such “stressful” situations can give rise to a condition which, due to its characteristics, severity, personal or professional frustration, or other specific circumstances, can presumably lead to damage, i.e., straining, even without proof of a precise persecutory aim [12].

When compared with mobbing, straining may result from even a single discriminatory action when the effect is permanent and existing (absence of systematicity)—whereas mobbing requires discriminatory actions to occur at least a few times a month for at least six months—and from a single type of action (absence of variability)—whereas mobbing requires at least two categories of hostile actions of the Leymann Inventory of Psychological Terrorism (LIPT) to occur. Therefore, mobbing creates a situation of conflict due to persecution, while straining generates a situation of forced stress due to discrimination [12].

Taking previous studies as a reference [42,54,55] and highlighting the link between straining and mobbing as the occurrence of negative actions causing devastating consequences for the employees, we decided to test the potential mediation effect of personality on the relationship between perception of straining at work and consequences in terms of health (physical and psychological) in a sample of HCWs.

Moreover, referring to literature results [54,56,57,58], this paper aims to investigate the role of some demographic variables (gender, age, marital status, schooling) and organizational variables (structure, area, position) in the emergence of a discriminatory conduct and its consequences.

Starting from the abovementioned observations, this research aims to verify the impact that straining actions have on the psychophysical health of workers and the role of personality in the interpretation of negative actions and in its impact on the psychophysical health of an individual.

In this vein, we designed a correlational study with a sample of HCWs to measure the following variables: straining perceptions and personality and individual consequences (psychological and physical). For the personality structure, we referred to the Big Five model [59,60] that identifies five principal personality factors (openness, neuroticism, extraversion, agreeableness, conscientiousness) and successfully used it to explore the impact of personality on behaviors, dispositions, and attitudes in different contexts [61,62,63].

**Figure 1:**
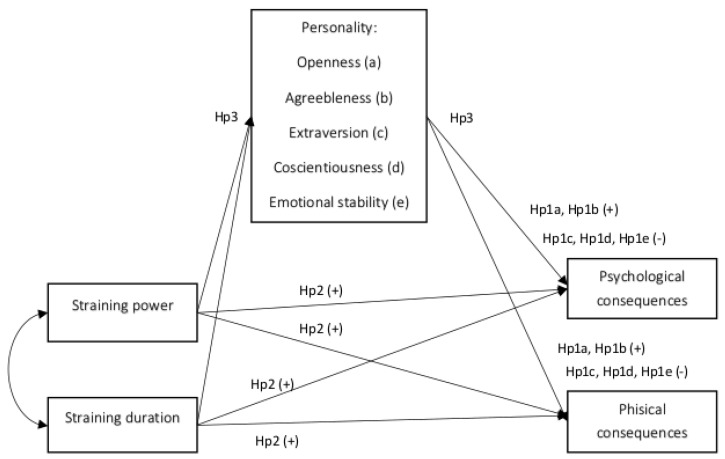
Research model and main hypotheses (Hp).

Extending the literature indications to the relationship between Big Five personality traits and behaviors at work [55,64,65,66], as well as to the effect of negative actions on health and personality [42,54], we designed a research model (see Figure 1) intended to verify the following hypotheses (Hp): Personality traits have an effect on individual consequences (Hp1); Since openness (Hp1a) and agreeableness (Hp1b) are expected to be positively related to individual consequences, extraversion (Hp1c), conscientiousness (Hp1d), and emotional stability (Hp1e) are negatively related to individual consequences (Hp1b); Negative act perceptions (personal bullying, social and work-related bullying) are positively related to individual consequences (Hp2a), while straining duration is negatively related to individual consequences (Hp2b).

More specifically, we wanted to test the hypothesis that personality has a mediation role in the relationship between perceptions of straining at work and individual consequences (Hp3).

Finally, we wanted to test the effect of demographical and job factors on S-NAQ, personality, and individual consequences (Hp4) (see Figure 1).

## 2. Materials and Methods

### 2.1. Sample

This research is part of a project aimed at evaluating organizational stressors and was commissioned by the peer departments of two public hospitals, co-partners of the project.

In this study, 416 (100%) HCWs of the two hospitals were invited by the management to complete a questionnaire during working hours, after attending a brief training about stress at work; 10% (n = 42) of them refused to participate in the survey, which, therefore, was administered to 374 workers (response rate 90%). Workers were invited by the researcher to participate on a voluntary basis and were informed about the aim of the study. All participants signed an informed consent form before taking part in the study.

Respondents were asked not to mention their name or the name of their organization anywhere in the questionnaire, in order to ensure privacy and anonymity. Data were collected in November 2018 in one hospital and in March 2019 in the other. The Internal Review Board (IRB) of the Department of Educational Sciences—University of Enna, Italy, approved the research (23/11/2018). All the data were processed according to the EU General Data Protection Regulation (GDPR).

Of the 374 HCWs, 55% (n = 207) were females, and 45% (n = 167) were males. Table 1 and Table 2 highlight the main sample and sub-samples characteristics. Age ranges: 51–60 (31%), 41–50 (24%), 31–40 (23%), <30 years (12%), and >60 (9%); 43% (n = 160) of HCWs had a degree; 56% (n = 210) of HCWs were married, and 26% (n = 96) were single; 65% (n = 243) of HCWs had children; 49% (n = 184) of HCWs were nurses, and 45% (n = 169) were physicians. The mean length of service was 18.9 ± 12.1 years; 81% (n = 302) of the participants declared to have a permanent contract, and more than half of them 74% (n = 276) were on shift work, with an approximate daily service of 7 h (7.1 ± 1.3 h).

### 2.2. Measures

Big Five Inventory—Italian Short Version (BFI-10) [67]. Personality was measured with a total of 10 items rated on a response scale ranging from 1 (completely disagree) to 5 (fully agree). Dimensions of the Italian BFI-10 are measured with 2 items each: agreeableness (AGR), conscientiousness (COS), emotional stability (EMS), extroversion (EXT), openness (OPEN). We re-phrased some items to reflect direct statements. An example is “Tends to find fault with others and does a thorough job”. Cronbach’s alpha = 0.85.

Short Negative Actions Questionnaire (S-NAQ)—Italian Version [68,69]. The S-NAQ is one of the most widely used instruments to evaluate any form of negative behavior [30,31,32,33,34,35,36,37,38,39,40,41]. All items in the NAQ-R scale are developed in behavioral terms, with no reference to bullying or harassment. The total score was intended as a measure of the power of the perceived straining. The number of suffered discriminatory actions was measured with a total of 9 items with a three-factor structure, namely, work-related bullying (WB), personal bullying (PB), and social bullyng (SB), rated on a 5-point response scale ranging from 1 (never) to 5 (always). Examples are, “They raised their voice” or “He was the target of instinctive rage attacks, and they have constantly criticized his work and his commitment”. Cronbach’s alpha = 0.91.

The perceived duration of the negative actions was intended as a measure of the straining duration and was assessed with 1 item, rated on a 9-point response scale ranging from 1 (never) to 9 (for over 5 years).

Occupational Stress Indicator—Italian Version [70,71]. Psychological consequences were assessed with the PSYT sub-scale of the Individual Consequences of Stress dimension of the Occupational Stress Indicator (OSI); 18 items were rated on a 6-point response scale ranging from 1 (completely false) to 6 (absolutely true). Examples of PSYT items are, “During the day, there are moments when you feel worried, upset, useful, confident”. We re-phrased some items to reflect direct statements. Cronbach’s alpha = 0.77.

Physical consequences were assessed with the PHIT sub-scale of the Individual Consequences of Stress dimension of the OSI; 18 items were rated on a 6-point response scale ranging from 1 (completely false) to 6 (absolutely true). Examples of PHIT items are, “Did you notice any symptoms over the last 6 months, such as lack of appetite, headache, or noise?”. The response scale ranged from 1 (hardly ever) to 6 (almost always). Cronbach’s alpha = 0.92.

Socio-Demographic Variables. Participants were asked to provide information on socio-demographic characteristics, such as gender, age, school grade, education, marital status, and work details, including type of contract (such as long-term or fixed-term contract), working hours (full time or part-time), work position, shift work, seniority, hours of service per day.

### 2.3. Data Analysis

In order to verify the hypotheses, correlation and regression analyses with SPSS 21.0 were conducted. Moreover, the research intended to test the hypothesis that personality has a mediation role in the relationship between perceptions about straining at work and individual consequences, using structural equation modeling analysis. Confirmatory factor analysis (CFA) with SEM via maximum likelihood estimation methods (along with the indicators’ covariance matrix) were used to evaluate the measurement and structural models concerning study variables and their associations, through AMOS 22 statistical package. With regard to the models’ goodness-of-fit evaluation, we relied on both absolute and relative goodness-of-fit indices. In addition, in order to refer to the chi-square and the degrees of freedom to evaluate possible significant differences between alternative nested models, we reported the following indices: root-mean-square error of approximation (RMSEA; acceptable values lower than 0.08;) [72] standardized root-mean-square residual (SRMR; acceptable values lower than 0.08;) [73] comparative fit index (CFI) and incremental fit index (IFI), for which scores higher than 0.90 are acceptable [74,75]. All study variables were measured through a single questionnaire; we addressed common method variance and response bias according to methods outlined by Podsakoff et al. [76]. Different scale endpoints and formats for the predictor and criterion measures were used in order to reduce method biases caused by commonalities in scale endpoints and anchoring effects. Moreover, we randomly inserted items into the questionnaire, and the scales were graphically separated from each other. Finally, two slightly different versions of the questionnaire, containing a different scales’ sequence (and mixed through the 2 different sub-samples), were used for data collection.

## 3. Results

To corroborate this research aimed at verifying the impact that straining actions have on the psychophysical health of a sample of HCWs and the role of personality in the interpretation of negative actions and in its impact on the psychophysical health of individuals, a confirmatory factor analysis was performed.

Dysfunctions of work organizations and the subsequent negative actions related to psychological and physical consequences in health care professionals are relevant concerns for the scientific community concerned with occupational health and safety, considering that, in Europe, this sector employs about 10% of the entire workforce [34].

In order to evaluate possible violations of the assumptions of normality, linearity, and multicollinearity, initial analyses (including Mahalanobis distance, and skeweness/kurtosis indices) were implemented (Table 3).

Table 4 reports the zero-order correlations among the variables of the study. The analysis of the different measurement models was conducted in order to assess the construct validity of the study measures using CFA.

Regarding the nine items of S-NAQ, CFA supported a three-factor—WB, PB, and SI—structure (χ^2^ = 79.21, df = 30, *p* < 0.001, χ^2^/df = 2.64, CFI (comparative fit index) = 0.940, GFI (goodness of fit index)= 0.946, SRMR = 0.038, RMSEA = 0.103, IFI = 0.941), and the composite reliability (CR) value was satisfactory (CR = 0.83).

CFA did not support the assumed five-factor structure of the 10-item BFI, because of its unsatisfactory model fit and its unacceptable factor loading of items of some specific scales (AGR and OPEN). Exploring other factor solutions of BFI with 10 items, a nearly acceptable result supported by CFA was a three-factor structure (χ^2^ = 161.7, df = 18, *p* < 0.001; GFI = 0.926; CFI = 0.908, TLI = 0.844, SRMR = 0.044, RMSEA = 0.097, IFI = 0.843), but with the AGR and OPEN items saturating on other expected factors and with not acceptable internal consistency (Cronbach’s alpha < 0.60). The best factor solution was highlighted by CFA for the one-factor solution of the 10-item BFI: χ^2^ = 79.21, df = 30, *p* < 0.001, χ^2^/df = 2.64; GFI = 0.938; CFI = 0.890, SRMR = 0.08, RMSEA = 0.071, IFI = 0.897), although it revealed a sufficient reliability (CR = 0.69). Considering the results, we proceeded to test measurement models with six items, excluding the OPEN and AGR sub-scales. CFA supported the assumed three-factor structure of the six-item BFI (EXT, COS, EMS), with an excellent model fit (χ^2^ = 31.17, df = 12, *p* < 0.001, χ^2^/df = 2.59; GFI = 0.989; CFI = 0.9670, SRMR = 0.08, RMSEA = 0.039, IFI = 0.942 and excellent factor loadings). The openness and agreeableness scales were excluded in the following analyses.

Confirmatory factor analyses revealed a two-factor structure for the OSI PSYT and PHIT consequences: χ^2^ = 1417.8, df = 559, *p* = 0.000, χ^2^/df = 2.53; GFI = 0.943; CFI = 0.910, SRMR = 0.062, RMSEA = 0.067, IFI = 0.902). Composite reliability was good (CR = 0.73).

### 3.1. Impact of Personality Factors on Psychological and Physical Consequences

The correlation analysis (see Table 4) clearly showed that only a few personality factors are related to psychological and physical consequences. Moreover, the regression analysis highlighted that emotional stability is the only Big Five subscale that clearly predicts both individual consequences (F (1, 374) = 42.90; *p* < 0.001; R^2^ = 0.103; ß = −0.268). This seems to stress that only specific personality factors have an impact on individual consequences. However, the assumed direction of the relationship between personality and consequences was confirmed for emotional stability (negative, Hp1e), conscientiousness (negative, Hp1d), and extraversion (negative, Hp1c). Overall, even if many of the sub-hypothesis were partially confirmed, the results corroborated the macro hypotheses Hp1 only for emotional stability; because of these results and because of the parallel lack of relationship with straining measures, only emotional stability was considered as a personality factor in the subsequent analysis.

### 3.2. Impact of Straining Perceptions on Psychological and Physical Consequences

The multiple regression analysis (see Table 5) highlighted that straining intensity predicts psychological (F (1, 374) = 28.09; *p* < 0.001; R^2^ = 0.098; ß = −0.281) and physical consequences (F (1, 374) = 73.24; *p* < 0.001; R^2^ = 0.17; ß = 0.544). Straining duration slightly predicted psychological consequences (F (1, 374) = 20.28; *p* < 0.001; R^2^ = 0.05; ß = −0.053) and physical consequences (F (1, 374) = 32.13; *p* < 0.001; R^2^ = 0.08; ß = −0.083). Analyzing the different sub-scales of S-NAQ with multiple regression analysis, the results highlighted that personal straining, compared to social and work straining, is the best predictor both for psychological consequences (F (1, 374) = 156.09; *p* < 0.001; R^2^ = 0.085; ß = 0.280) and physical consequences F (1, 374) = 26.99; *p* < 0.001; R^2^ = 0.17; ß = 0.407).

### 3.3. Impact of Socio-Organizational Variables on Psychological and Physical Consequences

The initial analysis also revealed that demographic and organizational variables (sex, age, tenure, and professional role) were not substantially related to the study variables, with the exception of gender (with males coded as 1, and females coded as 2).

Regression showed gender differences only for physical consequences: women reported significantly higher physical symptoms compared with men (t = −3.57, *p* < 0.001; physical consequences: men = 1.97; women = 2.33) assessed with the PHIT sub-scale of the Individual Consequences of Stress dimension of the OSI. Smaller subjective age differences in physical consequences (r = 0.133; *p* = 0.05) were found. The analysis underlined that schooling has a slight significant effect on both psychological and physical consequences.

### 3.4. Mediation Role of Personality

The Structural Equation Models (SEMS) allow to test the set of relationships described by a theoretical model through the analysis of the deviation or fit between the model supported by data and an ideal one.

In order to test if the effect of perceived straining on individual consequences is mediated by personality, starting from the initial model (Figure 1) and taking into account regression analysis, we developed a model (Model 1) with straining perceptions (intensity and duration) as independent variables and emotional stability and individual consequences as dependent variables.

The model contained all the possible relationships between the described model’s variables. Fit indices were not acceptable: χ^2^/df = 41.17; RMSEA = 0.327; CFI = 0.893; NFI (Normed Fit Index) = 0.893; NNFI (Non-Normed Fit Index) = 0.826; SRMR = 0.049.

**Figure 2:**
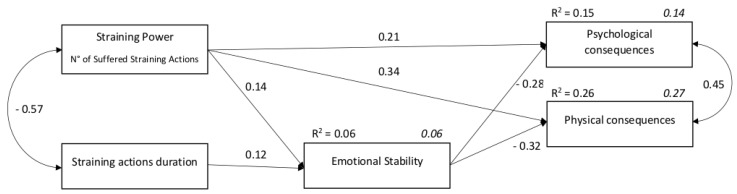
Path diagram of the tested model.

We then revised the model, removing non-significant relationships (straining duration on psychological and physical consequences) (Model 2). The fit indices were overall acceptable: RMSEA = 0.088; CFI = 0.924; NFI = 0.942; SRMR = 0.060; χ^2^/df = 14.3, *p* < 0.001. The path diagram is shown in Figure 2.

Overall, the effect of straining power on consequences was mediated by emotional stability (indirect β on psychological consequences = 0.21, *p* < 0.001; indirect β on physical consequences = 0.34, *p* < 0.001); the effect of straining duration on consequences was mediated by emotional stability (indirect β on psychological consequences = 0.08, *p* < 0.001; indirect β on physical consequences = 0.14, *p* < 0.001) (Table 6).

Thus, straining perceptions predicted emotional stability, which in turn determined better consequences. Straining explains only 8% of the variance of psychological consequences and 17% of the variance of physical consequences, while, together with emotional stability, it explains respectively 15% (for psychological consequences) and 26% (for physical consequences) of the variance. Altogether, SEM results seem to confirm that straining perceptions especially impact, through emotional stability, psychological consequences.

## 4. Discussion

The profound changes that have affected the occupational world in recent years have contributed to the emergence of new risk situations for the health and safety of workers [77,78,79,80].

In a highly precarious and highly conflictual work environment, workers’ situations of psychophysical discomfort tend to grow exponentially, undermining not only the physical integrity of a worker but, above all, his/her balance and emotional stability [81,82].

The focus of research on negative actions at work has progressively shifted from strictly organizational models to models that take into account dynamics and social interactions between workers, and subjective, inter-subjective, and cultural aspects that are involved in the processes of perception of straining, mobbing, and work-related stressors [12].

Straining is defined as a situation of attenuated mobbing, characterized by isolated but repeated actions over time that lead the worker to suffer constant stress and a progressive debasement of his/her working position [12,13].

The aim of straining is to marginalize the worker, often considered as an inconvenience or a hindrance to the career of the preferred people. The repercussions that such types of conduct produce on the subject are numerous and potentially very serious: from medium-level psychophysical disorders to more complex forms of depression and identity disorders.

However, few in the literature have investigated the effects of straining on health and the possible role of personality factors [10,41]. In this scenario, the role played by the personality factors of the straining victim is currently much debated and still far from being clarified, although it is the subject matter of many researches.

In general, research shows that personality factors can play the role of mediators in the relationship between the adverse situation experienced and the stress response, but that a specific profile of the mobbing victim cannot be described [10,30,33].

Generally, our study found support in other works for the research hypotheses and is in line with the scientific literature [27,83,84,85] regarding the identification of the variables that are mostly involved in degenerative events in the workplace [86].

Emotional stability has been examined and recognized as a personality representation in human mind [59]. Under the paradigm of the self-organizational theory, emotional stability indicates whether a complex emotional system can automatically maintain its equilibrium efficiently. It was suggested to include two dimensions: threshold of emotional response and emotional recovery from the methodology of self-organizations [60].

Threshold of emotional response represents the sensitivity of the emotional response, i.e., whether the individual experiences chaotic emotions (e.g., upset, anxiety, panic) easily. From the perspective of the evolutionary aspect, affect is an indicator attracting intentions on the adaptiveness of behaviors. Threshold of emotional response will determine whether an emotional system can be easily disordered [64].

The analyses conducted showed that HCWs are at risk of discriminatory actions. Straining intensity and duration predict psychological consequences and physical consequences. Our results highlighted that personal bullying, i.e., negative actions defined as attacks against the person (such as reputational aggressions), is the most frequent form of violence and the best predictor of both psychological and physical consequences to the HCWs who took part in this study; it tends to increase with the years of service (*p* = 0.012) and to consolidate the social and marital status (single, married).

Regarding the individual personality variables, conscientiousness is inversely proportional to WB, and agreeableness to PB. The effect of straining power on consequences is mediated by emotional stability. Emotional stability negatively correlates with the three component scales of S-NAQ: WB, PB, and SB. The role of the SB, as regards the correlation with outcomes in both the psychological and the physical health of HCWs, and the possible effect of mediation on the relationship between straining and consequences on psychological and physical health, appears, however, somewhat scaled down [87,88,89,90].

The results of this research should, in this sense, be considered in the light of some methodological limitations. This study is among the first to investigate the phenomenon of straining in healthcare professionals also in relation to personality traits. The references, in this case, were very poor and not intercountry, thus it was difficult to improve the discussion.

Data, in this sense, showed that only emotional stability is a personality factor that predicts individual consequences related to psychological and physical health. This seems to underline that only specific personality factors have an impact on individual consequences, and only emotional stability was considered in the subsequent analysis.

Some limitations need to be addressed in future research. The results should be interpreted with caution because of the type of sample and its size, which was not large enough; they may be generalized only to HCWs working in the same type of structure (public healthcare establishment). It will therefore be appropriate to extend this research to other types of workers and organizations. Drawbacks of the present study derive from getting results from a single sample, therefore they might not reflect attitudes and concern in other samples, even though the results are comparable to those observed in other surveys. Secondly, the information produced from this study is descriptive and correlational, and causation cannot be inferred.

Moreover, the personality measurement model did not provide the necessary reliability characteristics, and a significant part of the results might have been affected by this problem. In the future, it will certainly be useful to validate these results using samples from different work domains or to test differences between clinical (victims of straining) and non-clinical samples. The analysis of the different variables (organizational and personality) involved in straining and its effects must certainly be expanded through meticulous studies and tools. Future researches should better explore the role of personality (mediation, moderation, outcomes, etc.) in the perception of straining actions and in the effects on the individual.

Several indications in the literature have shown that, apart from socio-demographic factors and personality traits, circumstantial emotional states especially condition the perception of risk at work [78,79]. Some authors have described risk perception as a cognitive process mediated by an emotional component: emotions would represent heuristics (or mental shortcuts) that allow people to assess risks in a way that is certainly not rational, but functional [80,81,91]. Clearly, these temporary emotional states are mainly conditioned—and somehow managed—by our personality structure, but it will be the task of future research to clarify the weight of the different variables in the perception of negative actions and work-related stressors.

## 5. Conclusions

The discontinuous and flexible nature of contemporary work requires companies to develop forms of intervention aimed at the development of individual and interpersonal resources that are able to support workers in coping with situations of stress and difficulty at work.

When it comes to straining, as well as mobbing, part of the literature considers that the onset of many degenerative processes in the workplace is related to the personality traits of the victim [36,43,44,45]. This has led to a proliferation of works with a focus on the search for psychological traits typical of the victim and the aggressor [87,92,93]. As far as mobbing is concerned, for instance, several studies have identified MMPI, the Minnesota Multiphasic Personality Inventory profiles of victims [38,42], but only few papers have used Big Five profiles.

The overall results, in line with the scientific literature, show that HCWs suffer in the workplace especially in terms of personal discrimination, which has an impact on their psychophysical health. Comparing discriminating actions with personality traits, emotional stability, among all, correlates negatively with the three component scales of S-NAQ.

As to the practical consequences of this study, it seems clear that all these assumptions take on a remarkable role. Our results indicate that straining may cause major consequences also on job security, in terms of health and safety of workers and their performance. In managing straining behaviors, the management should implement training and monitoring policies that take account of individual differences, arranging ad hoc interventions and outlining a framework of risks that defines their borders within the company. In practice, the planning of straining risk interventions should focus on group activities that have a greater impact on individual behavior and, when they are able to increase the internal cohesion of work groups, can allow a greater prevention of conflicts and of the propensity for straining behaviors.

## Figures and Tables

**Table 1:** Description of the sub-samples.

		Gender		Profile			Corporate Seniority	Work Seniority
	N	Woman	Man	Doctor	Nurse	Staff	M (*SD*)	M (*SD*)
Hosp. A	207	100	107	78	87	42	21.37 (11.57)	15.59 (10.21)
Hosp. B	167	107	60	38	66	63	15.87 (11.95)	10.48 (10.04)
Total sample	374	207	167	116	153	105	18.9 (12.1)	12.97 (10.1)

**Table 2:** Description of the sample.

Age	*f* (%)	Marital Status	*f* (%)	Profile	*f* (%)
Up to 30 years	44 (11.8)	Unmarried	96 (25.7)	Doctors	169 (45.2)
31–40 years	87 (23.3)	Married	210 (56.1)	Nurses	184 (49.2)
41–50 years	91 (24.3)	Domestic partner	26 (7.0)	Technicians	21 (5.6)
51–60 years	117 (31.3)	Other	42 (11.2)	TOT	374 (100)
>61 years	35 (9.4)	TOT	374 (100)		
TOT	374				

**Table 3:** Descriptive statistics for different scales of the study. S-NAQ: Short Negative Acts Questionnaire.

	N° of Items	M (*SD*)	Skew.	Kurt.	Alpha
Extraversion	2	3.2 (0.87)	0.746	−0.641	0.921
Conscientious.	2	4.3 (0.84)	0.908	0.512	0.909
Agreeableness	2	2.9 (0.71)	−0.843	−0.772	0.720
Neuroticism	2	3.6 (0.91)	−0.521	0.895	0.936
Openness	2	3.2 (0.94)	−0.937	−0.719	0.731
S-NAQ power	9	1.6 (0.72)	0.466	−0.367	0.91
S-NAQ duration	1	5.8 (3.3)	−0.401	0.550	n/a
Psych. Conseq.	18	3.4 (0.76)	0.834	−0.767	0.77
Phys. Conseq.	17	2.1 (0.96)	0.722	0.511	0.92

**Table 4:** Zero-order correlations among the different scales of the study.

	M (±)	1	2	3	4	5	6	7	8
1 Extraversion	3.2 (0.87)								
2 Conscientious	4.3 (0.84)	0.162 **							
3 Agreeableness	2.9 (0.71)	−0.016	0.017						
4 Neuroticism	3.6 (0.91)	0.116 *	0.332 ***	0.051					
5 Openness	3.2 (0.94)	0.101 *	0.124 *	−0.143 **	0.013				
6 Strain (S-NAQ)	1.6 (0.72)	0.043	−0.091	−0.060	−0.235 **	0.046			
7 Durat. (S-NAQ)	5.8 (3.3)	−0.057	0.032	0.101	0.231 **	−0.088	−0.59 ***		
8 Psych. Conseq.	3.4 (0.76)	−0.18 **	−0.034	−0.165 **	−0.33 ***	0.132 *	0.275 **	−0.237 **	
9 Phys. Conseq.	2.1 (0.96)	−0.07	−0.132 **	−0.094	−0.39 ***	0.035	0.405 ***	−0.281 **	0.451 ***

* *p* < 0.05; ** *p* < 0.01; *** *p* < 0.001.

**Table 5:** Multiple regression of S-NAQ different sub-scales as predictor of health consequences.

	Psychological Consequences		Physical Consequences	
S-NAQ Factors	Beta (ß)	t	Beta (ß)	t
Work-related Bullying	−0.055	−0.580	0.057	0.638
Personal Bullying	0.354	3.861 ***	0.384	4.47 ***
Social Bullying	−0.021	−0.229	−0.01	−0.108
R^2^	0.098		0.17	

*** *p* < 0.001.

**Table 6:** Standardized path coefficient (regression weights).

			Estimate
Emot. Stab.	<---	Straining power	0.143 ***
Emot. Stab.	<---	Straining duration	0.124 **
Psych. Conseq.	<---	Straining power	0.214 ***
Physic. Conseq.	<---	Straining duration	0.078
Physic. Conseq.	<---	Straining power	0.341 ***
Psych. Conseq.	<---	Straining duration	0.069
Psych. Conseq.	<---	Emot. Stab.	−0.282 ***
Physic. Conseq.	<---	Emot. Stab.	−0.323 ***

** *p* < 0.01; *** *p* < 0.001.
